# A Frequency Up-Converted Hybrid Energy Harvester Using Transverse Impact-Driven Piezoelectric Bimorph for Human-Limb Motion

**DOI:** 10.3390/mi10100701

**Published:** 2019-10-15

**Authors:** Miah Abdul Halim, M. Humayun Kabir, Hyunok Cho, Jae Yeong Park

**Affiliations:** 1 Department of Electrical and Computer Engineering, University of Florida, Gainesville, FL 32601, USA; 2 Department of Electrical and Electronic Engineering, Islamic University, Kushtia 7003, Bangladesh; h.2011.kabir@gmail.com; 3 Department of Electronic Engineering, Kwangwoon University, Seoul 01897, Korea; whgusdhr@gmail.com (H.C.); jaepark@kw.ac.kr (J.Y.P.)

**Keywords:** transverse impact, frequency up-conversion, piezoelectric bimorph, human-limb motion, hybrid energy harvester

## Abstract

Energy harvesting from human-body-induced motion is mostly challenging due to the low-frequency, high-amplitude nature of the motion, which makes the use of conventional cantilevered spring-mass oscillators unrealizable. Frequency up-conversion by mechanical impact is an effective way to overcome the challenge. However, direct impact on the transducer element (especially, piezoelectric) increases the risk of damaging it and raises questions on the reliability of the energy harvester. In order to overcome this shortcoming, we proposed a transverse mechanical impact driven frequency up-converted hybrid energy harvester for human-limb motion. It utilizes the integration of both piezoelectric and electromagnetic transducers in a given size that allows more energy to be harvested from a single mechanical motion, which, in turn, further improves the power density. While excited by human-limb motion, a freely-movable non-magnetic sphere exerts transverse impact by periodically sliding over a seismic mass attached to a double-clamped piezoelectric bimorph beam. This allows the beam to vibrate at its resonant frequency and generates power by means of the piezoelectric effect. A magnet attached to the beam also takes part in generating power by inducing voltage in a coil adjacent to it. A mathematical model has been developed and experimentally corroborated. At a periodic limb-motion of 5.2 Hz, maximum 93 µW and 61 µW average powers (overall 8 µW·cm^−3^ average power density) were generated by the piezoelectric and the electromagnetic transducers, respectively. Moreover, the prototype successfully demonstrated the application of low-power electronics via suitable AC-DC converters.

## 1. Introduction

With the ongoing development of microelectronic technologies, multiple low-power consuming wireless sensor devices are being embedded within hand-held and wearable consumer electronics. These devices are mainly powered by an external power source (e.g., electrochemical batteries) of the consumer electronics and their continuous use allows it to run out of power quickly. Compared to device technologies, the development of the power sources (i.e., batteries) are still slower, even though the devices require less power to operate. Electrochemical batteries have a limited lifespan, and require periodic charging that is inconvenient or sometimes impossible. Moreover, since most batteries contain toxic chemicals, disposal of the expired batteries produces hazardous waste that enhances environmental pollution and poses threats to human and animal health. Therefore, there is great interest in developing self-powered electronics for uninterruptible and long-lasting operation by eliminating the need for recharging or replacing the power source. In recent years, energy harvesting from surrounding energy sources (e.g., light, heat, sound, vibration, etc.) has drawn much attraction to address these circumstances [1,2,3]. Among these sources, vibration is the most attractive physical energy source due to its versatility, incorruptibility, and abundance in nature [4]. However, different vibration sources (e.g., human and machine motion, water and wind flow, rotary motion etc.) generate vibrations of different frequencies and amplitudes, and mostly exhibit low-frequency, large-amplitude characteristics with various cyclic movements in different directions [5,6,7]. These vibrations, in the form of kinetic energy, can effectively be converted into electrical energy by employing compatible electromechanical transduction mechanisms that include piezoelectric [8], electromagnetic [9], electrostatic [10], magnetostrictive/magnetoelectric [11], and triboelectric [12] mechanisms. 

The performance of a vibration energy harvester greatly depends on the characteristics of vibration, the type of transducer, and how the transducer is coupled to the mechanical system. Generally, vibration energy harvesters utilize an inertial mechanism employed by a cantilevered spring-mass system, having a specific resonant frequency. Harvested energy (power) is at its maximum when the harvester’s resonant frequency matches the applied vibration frequency. Unfortunately, the power output decreases dramatically as the frequency of excitation (i.e., the resonant frequency of the harvester) decreases [13]. Moreover, employing a cantilevered spring-mass system for low-frequency (<10 Hz) energy harvesting is quite challenging due to the size constraints for specific application. Human-body-induced motion (e.g., walking, running, shaking limbs, etc.) also generates low-frequency (<6 Hz) vibrations, which do not allow the cantilever structure to be employed conveniently [14]. Hence, efficient energy harvesting from human-body-induced motion for hand-held and wearable smart devices requires clever design choices. Micro/nano-structured triboelectric nanogenerators [15,16], flexible piezo-composite based piezoelectric nanogenerators [17,18] etc. have shown great application potential in wearable biomechanical energy harvesting and motion sensing. However, they require huge efforts in material development, which was not of our interest. Our primary interest was to design and develop inertial based, low-frequency (e.g., human-body-induced motion) energy harvesters. 

The mechanical frequency up-conversion mechanism [19], among numerous design approaches over the past few years, has become the mainstream approach for human-motion based energy harvesting. It allows the transducer element (in the form of a spring-mass system) to actuate at its own resonant frequency (considerably high) by a low-frequency oscillatory or rotary system that responds to the external low-frequency vibration generated by human-motion. Commonly used methods of mechanical frequency up-conversion include mechanical impact and plucking [20,21,22,23]. Impact excitation transfers an instantaneous momentum into the transducer element whereas plucking excitation implies a slow deflection of the transducer element followed by its sudden release. In general, these methods exert direct force straight to the transducer element that could potentially lead to damage, especially in the case of piezoelectric devices. In order to overcome these issues with piezoelectric energy harvesters, we introduced the transverse impact-based frequency up-conversion mechanism in a human handy-motion driven electromagnetic energy harvester by employing a double-clamped FR4 cantilever beam as a high-frequency oscillator and a freely movable sphere as a low-frequency oscillator [24]. The transverse impact mechanism meets the reliability challenge and the freely-movable sphere allows the device to operate efficiently at extremely low frequencies (with sufficiently large amplitudes) of handy-motion vibration, meaning its non-resonant behavior [25]. However, the device generates low power and its average power density is poor. 

In order to improve its performance, we attempted to hybridize our previous work by incorporating a piezoelectric transducer without cost to the harvester volume. A hybrid energy harvesting technology combines two or more types of transducers that simultaneously capture energy from the same excitation [26,27,28]. In this paper, we present the theoretical modeling and experimental characterization of a piezoelectric (PE) and electromagnetic (EM) hybrid energy harvester for human-limb motion by utilizing the transverse mechanical impact-based frequency up-conversion strategy. Transverse impact, created by a sliding sphere over the parabolic tip of a mass attached to a clamped–clamped piezoelectric beam, eliminates the reliability issue from rapid damage of the piezoelectric cantilever due to direct impact. Moreover, simultaneous power generation from both PE and EM transducers offers a higher power density. A theoretical model for the hybrid generator under transverse impact was developed and experimentally validated with a prototype device. The proposed approach has the potential of reliable operation under low-frequency and high-amplitude excitation of human-body-induced motion toward the development of self-powered portable and wearable smart devices.

## 2. Design and Modeling 

### 2.1. Harvester Structure and Its Operation

**Figure 1:**
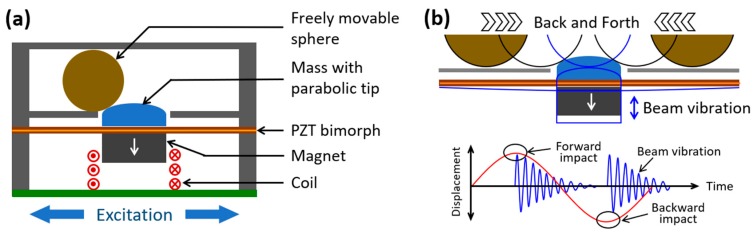
Schematics of the proposed transverse-impact driven frequency up-converted hybrid energy harvester (**a**) and its operation principle (**b**).

Figure 1a shows the schematic structure of the proposed transverse-impact driven hybrid energy harvester for human-limb motion. A PE transducer in the form of a clamped–clamped lead zirconate titanate (PZT) bimorph beam and an EM transducer consisting of one cylindrical magnet attached to the center of the piezo-beam and a hollow cylindrical multi-turn copper coil fixed to the housing constituted the hybrid generator structure. An additional mass with a parabolic-top was attached to the piezo-beam, opposite to the magnet. A hollow rectangular channel that contained a freely-movable spherical ball was placed on top of the piezo-beam in parallel. The channel had an opening at the center of its bottom wall that allowed the parabolic-top of the mass to be positioned through it, so that the ball was able to slide over the parabolic-top while the device was operated.

The principle of frequency up-conversion by transverse impact in the proposed hybrid energy harvester is illustrated in Figure 1b. When the device is excited by a low-frequency vibration with a sufficiently large amplitude (i.e., human-limb motion), the ball moves back and forth along the length of the channel. In its back and forth motion, the ball slides over the parabolic-top of the mass and produces a transverse impact on it that pushes mass as well as the piezo-beam downward, allowing it to vibrate freely at its own resonant frequency in a direction perpendicular to the direction of the ball movement. As a result, stresses are generated on the surfaces of the piezo-beam that generate voltage by virtue of the piezoelectric effect. Simultaneously, the magnet attached to the piezo-beam vibrates with respect to the adjacent coil and an electromotive force (e.m.f) voltage is generated by electromagnetic induction between them. The frequency (resonant) at which the beam vibrates is much higher than that of the excitation applied by human-limb motion and can be determined by the material and structural parameters of the beam. As shown in Figure 1b, the ball exerts transverse impact twice in one cycle of its back and forth motion; each time the system undergoes an impulse excitation, resulting in an exponentially decayed oscillatory motion between two consecutive impacts, so the output responses from both piezoelectric and electromagnetic transducers will be.

### 2.2. Electromechanical Modeling

The system is considered as a single-degree-of-freedom (SDOF) forced spring-mass-damper system excited by a periodic force F(t). When the ball slides over the parabolic-top of the cantilevered proof-mass, the collision between them is stated as the low-velocity transverse impact of a rigid body on a flexible element [20]. Accordingly, when the bodies (ball and proof-mass) come into contact, they tend to interpenetrate each other, and a local compression force develops in their interface, which increases as the ball slides over the parabolic-top of the proof-mass, resulting in bending of the beam. When the compression force is large enough, the ball slides over the parabolic-top before the force becomes large enough. However, the curvatures of both the ball and parabolic-top as well as the overlap between them plays crucial roles on the transverse impact mechanism. Finally, the bodies are separated and each vibrates independently until the next collision occurs. According to the force diagram of the transverse impact mechanism [20], the force experienced on the proof-mass in the transverse direction is $F\left(L,\;t\right)=\mu_{k}\;F\left(t\right)\;sin\theta$ and the governing equation of motion of the proposed system can be expressed as

(1) $$m\ddot{y}\left(t\right)+c\dot{y}\left(t\right)+ky\left(t\right)=\mu_{k}F\left(t\right)sin\theta \int_{0}^{L/2}\varphi \left(x\right)dx$$

where m is the mass (including the masses of the attached proof-mass and magnet); y(t) is the mass displacement; c is the equivalent damping coefficient; k is the stiffness of the beam; L is the length of the beam; $\mu_{k}$ is the coefficient of kinetic friction while the ball slides over the parabolic-top; and φ(x) is the mass normalized eigenfunction of the first vibration mode for the boundary condition x=L/2, which is [29]

(2) $$\varphi \left(x\right)=\sqrt{\frac{2}{m_{l}L}}\left[\cos h\frac{2\lambda }{L}x-\cos\frac{2\lambda }{L}x-\varsigma \left(\sin h\frac{2\lambda }{L}x-\sin\frac{2\lambda }{L}x\right)\right]$$

where $m_{l}$ is the mass per unit length; λ is the dimensionless frequency parameter for the first mode; and ς=(sinhλ−sinλ)/(coshλ+cosλ). 

**Figure 2:**
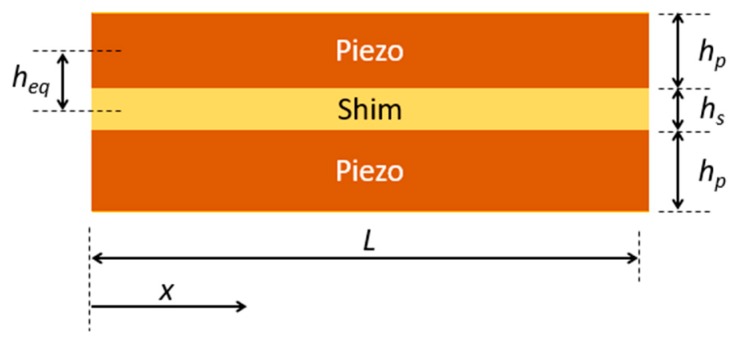
Cross-section of the piezoelectric bimorph beam.

The beam is a piezoelectric bimorph and generates voltage when lateral stress is generated on the surfaces of the piezoelectric material due to the transverse impact. The cross-section of the beam with a metallic shim sandwiched between two piezoelectric layers is shown in Figure 2. Each piezo-material is poled along its thickness direction and are connected in parallel. During bending of the beam, the stresses in the top and bottom piezoelectric layers will be in opposite directions: one is in tension and the other is in compression. An equivalent moment of inertia of the bimorph beam is defined as [30]

(3) $$I_{eq}=2\left(\frac{wh_{p}^{3}}{12}+wh_{p}h_{eq}^{2}\right)+\frac{E_{s}wh_{s}^{3}}{12E_{p}}$$

where $E_{s}$ and $E_{p}$ are the Young’s modulus of the shim and piezoelectric materials; $h_{s}$ and $h_{p}$ are the thickness of the shim and piezoelectric layers; and w and $h_{eq}\;\left(=\left(h_{s}+h_{p}\right)/2\right)$ are the width and the equivalent thickness of the beam, respectively. The maximum stress on the piezoelectric surface due to the transverse impact at the center of the beam (x=L/2) can be calculated as [31]

(4) $$\sigma_{max}=\frac{M\left(x\right)\left(h_{p}+\frac{h_{s}}{2}\right)}{I_{eq}}=\frac{\mu_{k}F_{max}L\left(h_{p}+\frac{h_{s}}{2}\right)}{8I_{eq}}$$

where M(x) is the bending moment and $F_{max}\left(=ky_{max}\right)$ is the magnitude of the transverse force determined by Hook’s law where k is the stiffness and $y_{max}$ is the maximum displacement of the bimorph beam. Now, the generated peak open circuit voltage can be determined as

(5) $$V_{oc}=\frac{-d_{31}h_{p}\sigma_{max}}{\varepsilon_{0}\varepsilon_{r}}$$

where $-d_{31}$ and $\varepsilon_{0}$ are the piezoelectric charge constant and dielectric constant of the piezoelectric material, respectively and $\varepsilon_{r}$ is the permittivity of free space. According to the dynamics of the transverse mechanical impact by the freely movable spherical ball described earlier, the output voltage from the piezoelectric transducer can be written as a function of time t as

(6) $$V_{PE}\left(t\right)=V_{oc}e^{-\zeta_{m}\omega_{r}t}sin\left(\omega_{d}t\right);\;n\frac{2\pi }{\omega_{d}}<t<\left(n+1\right)\frac{2\pi }{\omega_{d}},\;\left(n=0,1,2,3,\ldots \right)$$

where n is the number of impacts; $\omega_{r}$, $\omega_{d}$, and $\zeta_{m}$ are the resonant frequency, damped resonant frequency, and mechanical damping ratio, respectively, which are defined as [31,32]

(7) $$\omega_{r}=\sqrt{\frac{k}{m}}=\frac{\lambda }{L^{2}}\sqrt{\frac{E_{p}I_{eq}}{m}};\;\omega_{d}=\omega_{r}\sqrt{\left(1-\zeta_{m}^{2}\right)};\;\zeta_{m}=\frac{c_{m}}{2m\omega_{r}}$$

As the magnet attached to the piezoelectric beam also vibrates simultaneously, it induces voltage in the coil due to relative motion between them. According to Faraday’s law of electromagnetic induction, the induced open circuit e.m.f voltage generated by the electromagnetic transducer is [33]

(8) $$V_{EM}\left(t\right)=-N\frac{d}{dt}\left[\int \vec{B}.d\vec{A}\right]=-NBl\dot{y}\left(t\right)$$

where N is the number of coil turns and $\int \vec{B}.d\vec{A}$ indicates the net magnetic flux through the differential element area dA of the magnet-coil assembly. B is the magnetic flux density; l is the coil length across the magnetic flux lines; and $\dot{y}\left(t\right)$ is the relative velocity between the magnet and coil, which is determined by solving Equation (1) as

(9) $$\dot{y}\left(t\right)=-\frac{\mu_{k}F_{max}\omega_{r}\left[\int_{0}^{L/2}\varphi \left(x\right)dx\right]}{k\sqrt{1-\zeta_{m}^{2}}}e^{-\zeta_{m}\omega_{r}t}\sin\left(\omega_{d}t\right)$$

In the case of both transducers, the instantaneous power delivered to corresponding load resistance $R_{l}$ can be expressed as

(10) $$P\left(t\right)=\frac{1}{T}\int_{0}^{T}\frac{V{\left(t\right)}^{2}}{R_{l}}dt$$

It is to be noted that the damping $\left(\zeta_{T}\right)$ for each standalone (either piezoelectric or electromagnetic) transducer includes the mechanical damping $\zeta_{m}$ and the electrical damping $\zeta_{e}$ of the corresponding transducer: $\zeta_{T}=\zeta_{m}+\zeta_{e\;\left(PE\right)}$ for the piezoelectric transducer and $\zeta_{T}=\zeta_{m}+\zeta_{e\;\left(EM\right)}$ for the electro-magnetic transducer. However, for coupled transducers (when both transducers are terminated to corresponding loads simultaneously), the damping values are the same: $\zeta_{T}=\zeta_{m}+\zeta_{e\;\left(PE\right)}+\zeta_{e\;\left(EM\right)}$ for both transducers.

### 2.3. Simulation

Based on the above discussion on the electromechanical modeling of the proposed energy harvester, we performed time domain simulations using an appropriate simulation tool (MATLAB) to predict the output voltage generated by both PE and EM transducers simultaneously while operated by low-frequency excitation (i.e., human-limb motion). The parameters used in the simulation were calculated from the geometry and material parameters of the device components and will be discussed in the following sections. In the simulation, it was considered that the system was excited periodically in the horizontal direction at 5 Hz frequency and 2 g (g = 9.8 ms^−2^) peak acceleration. It was also assumed that the sphere started moving from the left end of the rectangular channel and moved back and forth periodically in response to the applied excitation. 

**Figure 3:**
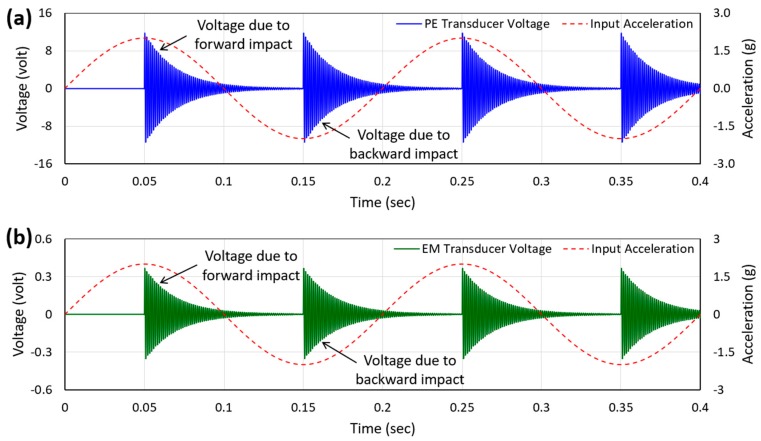
Simulated open circuit voltage waveforms of the piezoelectric (**a**) and electromagnetic (**b**) transducers at 5 Hz excitation frequency and 2 g peak acceleration.

Figure 3 shows the simulated open circuit voltage waveforms generated by the PE and EM transducers. In each voltage waveform, two consecutive maximum peaks occurred due to the transverse impact when the sphere slid over the proof-mass during its forward and backward motion in one cycle and the process continued as long as the excitation existed. The positive half cycle of the acceleration waveform indicates the forward motion and the negative half cycle indicates the backward motion of the sphere. Since both transducers generated voltage simultaneously, the frequency of both open circuit voltage waveforms was the same, which was the resonant frequency of the vibrating piezoelectric beam. As seen in the figure, the amplitudes of the instantaneous voltage waveforms (in both cases) decayed exponentially with time due to mechanical damping and became almost zero until the next impact occurred.

## 3. Prototype and Test Setup 

### 3.1. Prototype Fabrication

**Figure 4:**
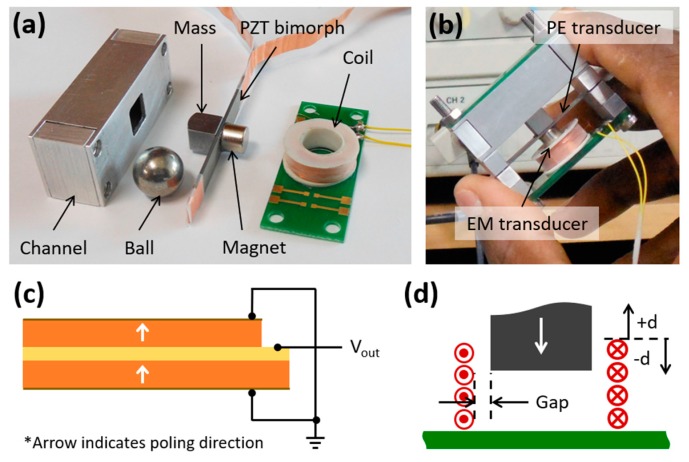
Photographs of the prototype components (**a**), fabricated prototype (**b**), schematics of the parallel bimorph connection (**c**), and the magnet-coil assembly (**d**).

A macro-scale prototype of the proposed hybrid energy harvester was fabricated and tested. The PE transducer of the prototype comprised a piezoelectric (PZT) parallel bimorph (SMBA4510T05M, STEMiNC, Davenport, FL, USA), a neodymium (N52) cylinder magnet, and a cubic iron mass of 6 mm length with a 1 mm high parabolic top, both glued to the middle of either sides of the bimorph beam. A suitable assembly of a cylinder magnet and a 1000-turn coil (0.1 mm diameter laminated copper wire) attached to a printed circuit board (PCB) constituted the EM transducer. A 316 stainless steel ball was enclosed in a rectangular shaped aluminum channel (inner area 10.5 × 10.5 mm^2^) with a square (7 × 7 mm^2^) opening at the middle of its bottom wall, which was assembled on top of the PE transducer. The channel opening was occupied by the parabolic-top of the cubic mass with a 0.4 mm overlap with the ball. Figure 4 shows a photograph of the fabricated prototype device along with the schematics of the electrical connection of the piezoelectric bimorph and the magnet-coil assembly. The geometric parameters and material properties of the components are tabulated in Table 1.

### 3.2. Human-Limb Motion Test Setup

Our fabricated energy harvester was tested by human-limb motion to observe its power generation capability under a real-world situation. In order to achieve a robust test setup, it required convenient (small and portable) measuring equipment to record the characteristics (frequency and amplitude) of the excitation generated by human-limb motion. An EVAL-ADXL326Z (Analog Devices Inc., Norwood, MA, USA) tri-axial MEMS accelerometer kit (mounted on the harvester prototype) in conjunction with a XR5-SE (Pace Scientific Inc., Mooresville, NC, USA) data logger was used to record the excitation profile of human-limb motion for further analysis. The outputs of both PE and EM transducers were connected to a digital storage oscilloscope (TDS 5052B, Tektronix Inc., Beaverton, OR, USA) to observe and record the output responses. Furthermore, benchtop tests using an electrodynamic shaker were conducted to observe the damping behavior of both transducers and to determine the optimal overlap (±d) between the magnet and coil, as described in our previous work [24].

## 4. Experimental Results and Discussion

### 4.1. Optimal Overlap and Damping Measurements

**Figure 5:**
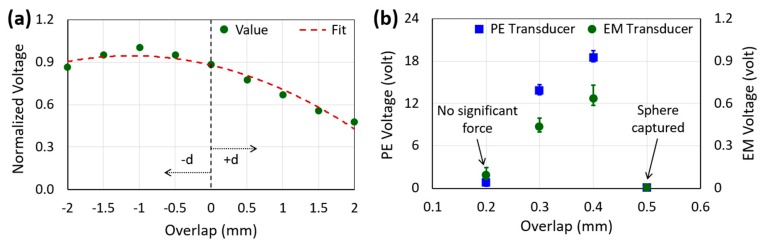
Normalized open circuit voltages for different magnet-coil overlaps (**a**) and open circuit voltages of the transducers for various overlaps between the freely movable sphere and the parabolic top of the proof-mass (**b**).

In order to generate maximum possible voltage and power from the prototype, it was important to determine the optimum overlaps between the magnet and coil as well as between the freely-movable sphere and parabolic-top of the proof-mass. The optimum magnet-coil overlap was determined by a benchtop test setup [24] using an electrodynamic shaker whereas the overlap between the sphere and the mass-top was determined by the human-limb vibration test setup (due to the limitation of the shaker to generate low-frequency, large-amplitude excitation). As seen from Figure 5a, the optimum magnet-coil overlap was −1 mm. The lateral gap between the magnet and coil was also 1 mm. Since the absolute values were not primarily of interest in determining the optimum magnet-coil overlap, normalized values were used. Figure 5b shows the change in the open circuit voltages generated by both the PE and EM transducers with the change in the overlap between the sphere and the mass-top. As seen from the figure, the sphere could not make significant contact with the parabolic top when the overlap was 0.2 mm as the clearance between the ball and inner surface of the channel was 0.2 mm. On the other hand, the sphere could not slide over the mass-top and was captured in the middle when the overlap was 0.5 mm because the speed/force of the sphere was not sufficient to pass through. The 0.4 mm overlap between the sphere and mass-top was considered as the optimum value since the open circuit voltages were the maximum for both the PE and EM transducers. The error bars in Figure 5b indicate the range of voltages generated for multiple attempts as the characteristics of the excitation (frequency and amplitude) applied by human-limb were not always the same. 

The damping behavior of both PE and EM transducers were determined by the impulse response test using an electrodynamic shaker [24]. A high amplitude impulse (30.3 ms^−2^ with 50 ms pulse period and 500 µs pulse width) was applied to the harvester. Then, the mechanical damping ratio ($\zeta_{m}$) and total damping ratio ($\zeta_{T}$) of both transducers were estimated from the open circuit and loaded impulse response signals, respectively. The logarithmic decrement method was used to calculate the damping ratio as

(11) $$\zeta =\frac{1}{2\pi }\ln\left(\frac{a_{1}}{a_{2}}\right)$$

where $a_{1}$ and $a_{2}$ are the amplitudes of two consecutive peaks in the impulse response plot of the transducer. Subtraction of the mechanical damping ratio ($\zeta_{m}$) from the total damping ratio ($\zeta_{T}$) gives the electrical damping ratio ($\zeta_{e}$). By conducting this experiment, the mechanical damping ratio was found to be 0.011. On the other hand, the electrical damping ratio for the piezoelectric transducer and electromagnetic transducer were 0.017 and 0.016, respectively. It should be noted that the electrical damping values were determined by the impulse response across the corresponding optimum load resistances of the transducers, which were determined by measuring the voltage across various load resistors and calculating the power delivered to them. The power is experimentally equal to $V_{p-p}^{2}/4R_{l}$, where $V_{p-p}$ is the peak–peak value of the measured voltage across each load resistance $R_{l}$. 

### 4.2. Transducer Outputs

**Figure 6:**
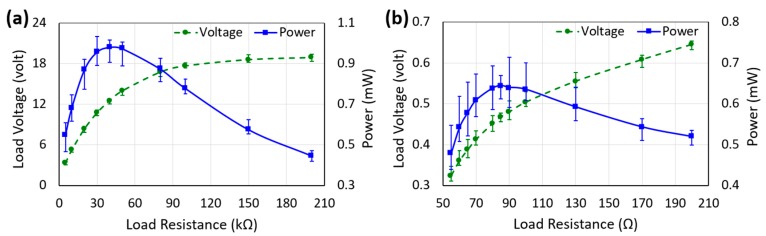
Measured voltage and power vs. load resistances connected to the piezoelectric (**a**) and electromagnetic (**b**) transducers while excited by human-limb motion.

**Figure 7:**
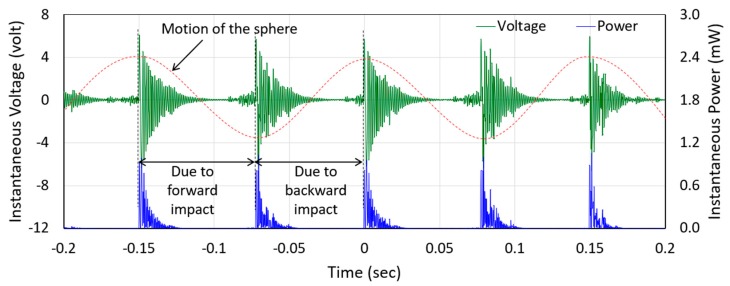
Instantaneous voltage and power waveforms measured across the 40 kΩ optimum load resistance of the piezoelectric transducer during the human-limb motion test.

Figure 6 illustrates the measured peak–peak voltages and peak powers delivered to the load resistances connected to the PE and EM transducers, while the prototype was excited by human-limb motion. Error bars indicate the range of voltage and power values measured for multiple attempts. On average, the maximum of 0.98 mW and 0.64 mW peak power were delivered to 40 kΩ and 85 Ω load resistances connected to the PE and EM transducers, respectively. Note that the optimum load resistances for the PE and EM transducers were in the range of kΩ and Ω, respectively. The resistance of the coil was measured as 84 Ω, which closely matches that of the measured optimum load of 85 Ω. On the other hand, the source resistance (*R*_source_) of a piezoelectric material depends on its vibration frequency (*f*) and capacitance (*C*), according to *R*_source_ *=* 1/(2π*fC*). The capacitance of the piezoelectric beam (doubly clamped parallel bimorph) was measured as 16 nF and the frequency of its vibration was calculated as 815 Hz (measured as 818 Hz). This gives the calculated *R*_source_ as 38.3 kΩ, which closely matches the measured optimal load resistance of 40 kΩ. Figure 7 shows the instantaneous voltage and power waveforms across 40 kΩ optimum load resistances of the PE transducer. The voltage and power waveforms generated by the EM transducer also followed the same trend. The maximum peak–peak voltages across the corresponding optimum load resistances generated by the PE and EM transducers were 12.53 V and 0.47 V, respectively. However, the peaks of both voltage and power waveforms decayed exponentially with time due to the damping, which, in turn, reduced their rms (1.92 V for PE and 72 mV for the EM transducers) and average values (93 µW for the PE and 61 µW EM transducers), respectively. Peak amplitudes of the instantaneous power were reduced to almost zero as the time passed, and before the next impact occurred. As a result, the values of average power reduced dramatically. It is to be noted that the waveforms were collected simultaneously, therefore, the overall damping was composed of mechanical damping and the electrical damping of both transducers, as discussed earlier. As seen from the figure, the amplitude decays were not perfectly exponential due to process variation in assembling the harvester components. Two consecutive maximum peaks were generated in one cycle of the applied excitation since the sphere exerted transverse impact on the mass-top twice during its back and forth movement in one cycle. It should be noted that there was no significant change in the peak values of the voltage and power with the change in the frequency of human-limb motion as the variation in the acceleration amplitude was small, however, the values of the rms voltage and average power output changed with the change in the frequency of excitation [34]. This occurred because of the change in the time interval between two consecutive impacts with the change in the frequency and was also due to the exponentially decaying behavior of the voltage waveform generated by the transducer.

**Figure 8:**
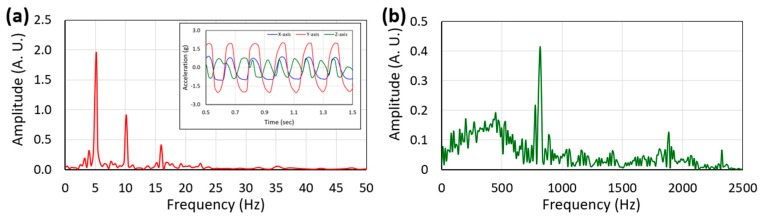
Frequency components (FFT) of the applied excitation (along Y-axis) obtained from the accelerometer data (inset) during the test (**a**) and the voltage waveform generated by the piezoelectric transducer (**b**).

The input excitation characteristics (frequency and amplitude of human-limb motion) were measured along each axis of the accelerometer mounted on the prototype during the test. As the harvester prototype was driven along the accelerometer’s Y-axis, the peak acceleration amplitude was maximum in this direction (~2 g), whilst those in other directions were relatively low (~0.95 g along X-axis and ~0.75 g along Z-axis). Data were collected at the 50 Hz sampling rate. The frequency components of both applied acceleration and the generated voltage waveforms were determined by Fast Fourier Transform (FFT) analysis. Figure 8 shows that the frequency of the applied excitation was 5.2 Hz whereas the frequency of the voltage waveform generated by the PE transducer (same for the EM transducer) was 818 Hz, indicating the frequency up-conversion behavior of the harvester.

### 4.3. AC–DC Conversion

**Figure 9:**
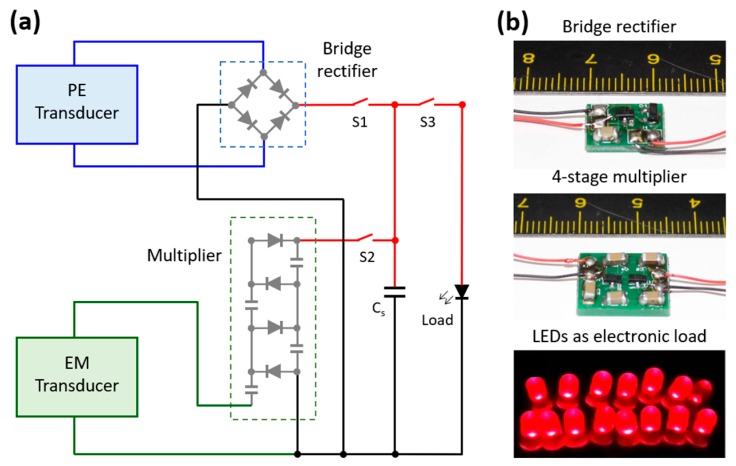
Schematic of the hybrid energy harvester circuit diagram (**a**), photographs of a bridge rectifier, 4-stage voltage multiplier as the AC–DC converters and LEDs powered by the harvester as electronic load (**b**).

The voltage generated by the proposed harvester has alternating (AC) characteristics due to the time-varying characteristic of the input excitation. Most electronic devices are driven by DC voltage source. Therefore, AC–DC conversion is necessary before using the harvested energy. Generally, a full bridge rectifier using four diodes is used to rectify the ac voltage generated by the harvester unit. In our prototype harvester, the voltage generated by the EM transducer was very low when compared to that of the PE transducer. Therefore, a conventional bridge rectifier cannot satisfy the need for rectification and significant voltage generation to drive an electronic load. This is why, a 4-stage Villard’s voltage multiplier circuit was used with the EM transducer whereas a bridge rectifier, on the other hand, was used with the PE transducer for AC–DC conversion, as shown in Figure 9a. The voltage multiplier rectifies the voltage output with voltage multiplication based on the number of stages used [35]. The bridge rectifier used four Schottky barrier diodes whereas the voltage multiplier circuit used four pairs of Schottky barrier diodes (HSMS-2852-BLKG, Broadcom Inc., San Jose, CA, USA) and 10 µF, 50 V capacitors, soldered on a printed circuit board (PCB) designed by a professional PCB design tool (Proteus 8.0). The outputs of both bridge rectifier and multiplier circuit were connected to a 33 µF, 50 V storage capacitor (C_s_) to accumulate the rectified and multiplied DC electrical energy that was used to power a number of parallelly connected LEDs that demonstrated its application potential, as shown in Figure 9b.

**Figure 10:**
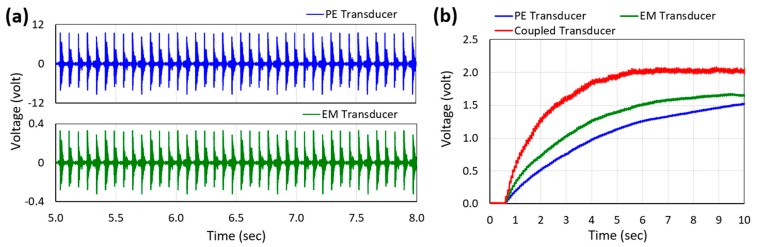
Voltage waveforms generated by each transducer (**a**) and accumulated rectified and multiplied voltages across the storage capacitor (**b**) as a function of time while excited by human-limb motion.

Figure 10a shows the output AC voltage waveforms of the PE and EM transducers of the prototype harvester (with the rectifier and multiplier connected) while excited by human-limb motion, to be converted to DC and stored in the storage capacitor (C_s_). The charging characteristics of the storage capacitor (C_s_) was also observed at the same time, as presented in Figure 10b. The charging behavior is influenced by the inherent output characteristics (voltage and current) of the piezoelectric and electromagnetic transducers where the voltage determines the maximum limit of charging and the current determines the charging speed. As a result, the high output current and low output voltage of the electromagnetic transducer charges the capacitor relatively faster than the low output current and high output voltage of the piezoelectric transducer. When the DC outputs from both transducers were coupled together, the storage capacitor was charged even faster and reached over 2 V DC voltage and was able to turn on the LEDs used as the electronic load.

## 5. Conclusions and Future Works

This paper presents a human-limb motion driven, piezoelectric and electromagnetic hybrid energy harvester that utilized the frequency up-conversion technique by the transverse impact mechanism. Instead of using any resonant structure (e.g., compliant cantilever beam), a freely movable non-magnetic metallic sphere was used as the low-frequency oscillator, which overcomes the limitations of designing energy harvesters for human-body-induced motion. Use of two transducers allows simultaneous power generation from a single mechanical excitation, which increases the power density of the harvester. The theoretical model was derived based on its working principle, and then a macroscale prototype was fabricated and tested. A series of tests were carried out to partially optimize its parameters and to observe its output performances. The piezoelectric and electromagnetic transducers of the prototype energy harvester simultaneously generated maximum 93 µW and 61 µW average powers, respectively, while excited by human-limb motion at ~2 g peak acceleration. Analysis of the measured voltage and acceleration data shows that the frequency was up-converted to 818 Hz from 5.2 Hz human-limb motion. In order to utilize the harvested energy for practical low-power electronics applications, suitable AC–DC converters (rectifier for PE and voltage multiplier for EM transducers) were constructed and demonstrated. For a functional volume of 19.2 cm^3^, the average power density of the hybrid energy harvester prototype was 8 µW cm^−3^, which is ~1.5× higher than its electromagnetic only counterpart (5.4 µW cm^−3^). However, the generated power and the power density of the harvester was still low as the size, mass, and diameter of the ball, height and curvature of the attached mass-top, stiffness of the piezoelectric beam, etc. were chosen arbitrarily, which are all significantly related to power generation. Further optimization of these parameters would be able to deliver higher power within a reduced volume. A more portable design and lighter packaging material should be adopted for its intended use. Our future work will include further optimization of the design parameters (e.g., spring stiffness, mechanical and electrical damping, transverse impact, magnet-coil assembly, etc.) through finite element analysis (FEA) tools, and to fabricate a compact and smaller device with improved output performances to be efficiently used in powering portable and wearable smart devices from human-body-induced motion.

## Figures and Tables

**Table 1:** Geometric parameters and materials properties of the harvester components.

Parameter	Value
Dimension of the Piezoelectric bimorph	40 × 6 × 0.5 mm^3^
Thickness of each piezoelectric (PZT) layer	0.2 mm
Thickness of middle shim (copper) layer	0.1 mm
Young’s modulus of PZT	72 GPa
Dimension of the cylinder magnet	Ø6 × 5 mm^2^
Remnant flux density of the magnet	1.18 T
Mass of the magnet	1 g
Mass of the attached proof-mass	1.73 g
Diameter of the sphere	10.3 mm
Mass of the sphere	4.36 gm
Length of the channel	30 mm
Inner diameter of the coil	8 mm
Outer diameter of the coil	10 mm
Number of coil turns	1000
Height of the coil	5 mm
Resistance of the coil	84 Ω
Dimension of the fabricated prototype	40 × 30 × 16 mm^3^
